# Storage protein profiles in Spanish and runner market type peanuts and potential markers

**DOI:** 10.1186/1471-2229-6-24

**Published:** 2006-10-12

**Authors:** XQ Liang, M Luo, CC Holbrook, BZ Guo

**Affiliations:** 1 USDA-ARS, Crop Protection and Management Research Unit, Tifton, GA, USA; 2 Guangdong Academy of Agricultural Sciences, Institute of Crop Sciences, Guangzhou, China; 3 University of Georgia, Department of Crop and Soil Sciences, Tifton, GA, USA; 4 USDA-ARS, Crop Genetics and Breeding Research Unit, Tifton, GA, USA

## Abstract

**Background:**

Proteomic analysis has proven to be the most powerful method for describing plant species and lines, and for identification of proteins in complex mixtures. The strength of this method resides in high resolving power of two-dimensional electrophoresis (2-DE), coupled with highly sensitive mass spectrometry (MS), and sequence homology search. By using this method, we might find polymorphic markers to differentiate peanut subspecies.

**Results:**

Total proteins extracted from seeds of 12 different genotypes of cultivated peanut (*Arachis hypogaea *L.), comprised of runner market (*A. hypogaea ssp. hypogaea*) and Spanish-bunch market type (*A. hypogaea ssp. fastigiata*), were separated by electrophoresis on both one- and two-dimensional SDS-PAGE gels. The protein profiles were similar on one-dimensional gels for all tested peanut genotypes. However, peanut genotype A13 lacked one major band with a molecular weight of about 35 kDa. There was one minor band with a molecular weight of 27 kDa that was present in all runner peanut genotypes and the Spanish-derivatives (GT-YY7, GT-YY20, and GT-YY79). The Spanish-derivatives have a runner-type peanut in their pedigrees. The 35 kDa protein in A13 and the 27 kDa protein in runner-type peanut genotypes were confirmed on the 2-D SDS-PAGE gels. Among more than 150 main protein spots on the 2-D gels, four protein spots that were individually marked as spots 1–4 showed polymorphic patterns between runner-type and Spanish-bunch peanuts. Spot 1 (ca. 22.5 kDa, pI 3.9) and spot 2 (ca. 23.5 kDa, pI 5.7) were observed in all Spanish-bunch genotypes, but were not found in runner types. In contrast, spot 3 (ca. 23 kDa, pI 6.6) and spot 4 (ca. 22 kDa, pI 6.8) were present in all runner peanut genotypes but not in Spanish-bunch genotypes. These four protein spots were sequenced. Based on the internal and N-terminal amino acid sequences, these proteins are isoforms (iso-*Ara *h3) of each other, are iso-allergens and may be modified by post-translational cleavage.

**Conclusion:**

These results suggest that there may be an association between these polymorphic storage protein isoforms and peanut subspecies *fastigiata *(Spanish type) and *hypogaea *(runner type). The polymorphic protein peptides distinguished by 2-D PAGE could be used as markers for identification of runner and Spanish peanuts.

## Background

There is considerable variation in *Arachis hypogaea *L. subspecies *hypogaea *and *fastigiata *Waldron, which are further classified into four market types including runner, Virginia, Spanish, and Valencia [1]. Most cultivated peanuts belong to Spanish and runner types. They exhibit genetically-determined variation for a number of botanical and agronomical traits including branching and flowering habits, seed dormancy, and maturation time. However, there are few categorical criteria for distinguishing subspecies because of the limited detectable molecular polymorphism. Recently, several molecular approaches have been employed to assess genetic diversity and taxonomic relationships. Among them are isozymes [2], restriction fragment length polymorphisms (RFLP), random amplified polymorphisms (RAPD), amplified fragment length polymorphisms (AFLP), and simple sequence repeats (SSR) [3-6]. However, very little genetic polymorphism between the two subspecies was detected. Singh et al. [7,8] and Bianchi-Hall et al. [9] found very limited or no variation among cultivated peanut based on seed protein profiles.

To date, proteomic analysis has proven to be the most powerful method for describing plant species and lines [10], and identification for proteins (especially protein markers) in complex mixtures. The strength of this method resides in high resolving power of two-dimensional PAGE (2D-PAGE), coupled with polypeptide sequencing by highly sensitive mass spectrometry (MS) such as electrospray ionization tandem mass spectrometry (ESI-MS/MS), and sequence homology search in databases [11].

The aim of the research described in this paper was to investigate the ability of proteomic analysis to assess diversity of seed storage proteins in peanut for subspecies or cultivar identification. Subspecies or cultivar-specific proteins, if they exist, should be helpful for genetic studies, breeding, taxonomy and evolutionary relationships in peanut.

## Results

### Analysis of gel electrophoresis

**Figure 1:**
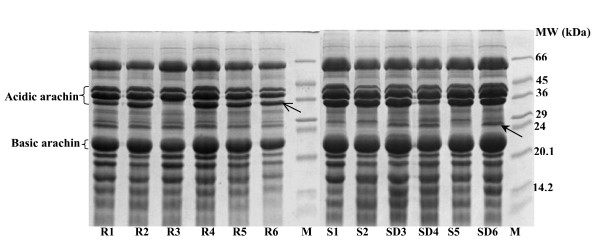
**SDS-PAGE peanut seed total protein profiles**. One-dimensional SDS-PAGE of peanut seed protein of runner (R) and Spanish (S) or Spanish derivatives (SD): R1 = A104, R2 = GK 7, R3 = A13, R4 = Tifrunner, R5 = A100, R6 = Georgia Green; S1 = ICGV 95435, S2 = MXHY, SD3 = GT-YY7, SD4 = GT-YY79, S5 = ZQ 48, SD6 = GT-YY20; M = molecular weight standards. The arrow () indicates the protein band with a molecular weight of 35 kDa and the arrow () indicates the 26 kDa protein band.

Total protein extracts from six runner and six Spanish-bunch peanut cultivars and lines were separated by one-dimensional SDS-PAGE, and the protein profiles revealed few major difference among all tested peanut genotypes (Fig. 1). Proteins were resolved as four groups (conarachin, acidic arachin, basic arachin, and smaller than 20 kDa). All but one peanut genotype had three strong bands in the range of 35 to 45 kDa, which corresponds to acidic arachins. Runner peanut A13 did not have this 35 kDa polypeptide, a subunit of *Ara *h3 present in other genotypes. This 35-kDa protein peptide was reported as a 36-kDa protein associated with blanchability in peanut [12]. A polymorphic protein band with a molecular weight of about 26 kDa were present in all six runner type genotypes and three Spanish derivatives GT-YY7, GT-YY79, and GT-YY20, which all have a runner type peanut, Induhuanpi, in their pedigrees (Fig. 1).

**Figure 2:**
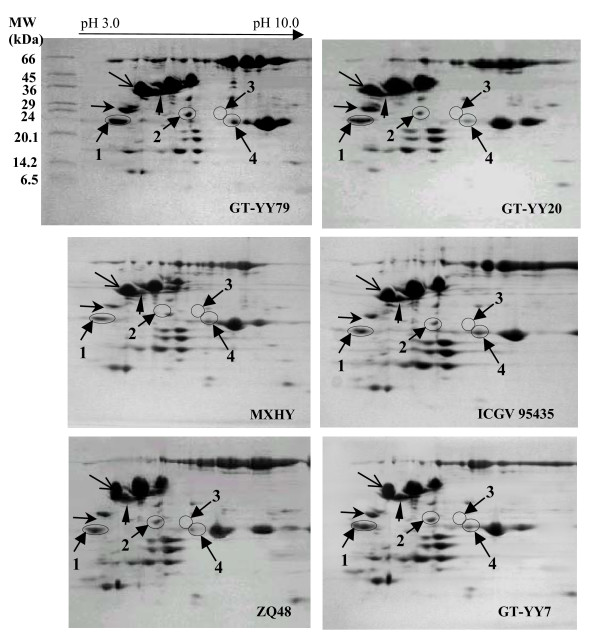
**2-D SDS-PAGE peanut seed total protein profiles**. Two-dimensional SDS-PAGE of peanut seed total protein profiles of 6 cultivated peanut genotypes, Spanish market type. Gels are oriented with the acid end of the isoelectric focusing separating to left and the basic end to the right. The arrow () indicates the protein band with a molecular weight of 35 kDa and the arrow () indicates the 27 kDa protein band (Fig. 1). The arrow head () indicates the fourth band as reported for Spanish cultivars [9]. The numbered arrows () pointing to cycled spots indicate the polymorphic polypeptide spots, which were sequenced (Table 2).

**Figure 3:**
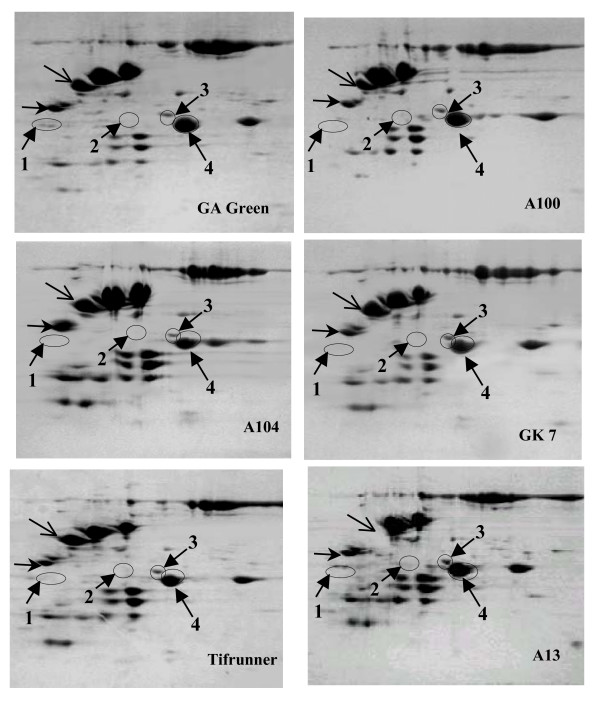
**2-D SDS-PAGE peanut seed total protein profiles**. Two-dimensional SDS-PAGE of peanut seed total protein profiles of 6 cultivated peanut genotypes, runner market type. Gels are oriented with the acid end of the isoelectric focusing separating to left and the basic end to the right (Fig. 2). The arrow () indicates the protein band with a molecular weight of 35 kDa and the arrow () indicates the 27 kDa protein band (Fig. 1). The numbered arrows () pointing to cycled spots indicate the polymorphic polypeptide spots, which were sequenced (Table 2).

We used two-dimensional electrophoresis (2-D PAGE) to achieve a better protein profile of each genotype (Fig. 2 and Fig. 3). Total protein from 12 peanut cultivars or breeding lines was subjected to 2-D PAGE, resulting in about 150 spots found in all cultivars. These protein peptide spots covered a range of isoelectric points (p*I*s) (pH 3–10) and molecular masses (10 – 66 kDa). Many components that were recorded on SDS-PAGE gel as a single band (Fig. 1) were resolved into several distinct spots with different pI values by 2-D PAGE gels (Fig. 2 and Fig. 3). The conarachin group (*Ara *h1) with about 65 kDa molecular weight by SDS-PAGE was separated into many spots with different p*I*s. Interestingly, the acidic arachin group with three clear bands ranging from 35 – 45 kDa for all genotypes but A13 (Fig. 1) was resolved into two bands by SDS-PAGE. There was additional polymorphism on 2-D PAGE showing an additional spot in Spanish type peanut as indicated by a arrow head (Fig. 2), which confirmed the report by Bianchi-Hall et al. [9]. The 35 kDa and 26 kDa protein bands, revealed on SDS-PAGE, were confirmed on 2-D PAGE. The basic arachin group with one heavy band on SDS-PAGE at about 22 kDa was separated into several spots or subunits on the 2-D PAGE with distinct isoelectric points and slight differences in molecular weights (Fig. 2 and Fig. 3). These patterns revealed polymorphisms between runner type and Spanish type genotypes. There were four distinct protein spots labelled as spots 1–4. Spot 1 (ca. 22.5 kDa, p*I *3.9) and spot 2 (ca. 23.5 kDa, p*I *5.7) were observed in all Spanish-bunch genotypes, but were not found in those of runner types. In contrast, spot 3 (ca. 23 kDa, p*I *6.6) and spot 4 (ca. 22 kDa, p*I *6.8) were present in all runner genotypes but spot 3 was not in Spanish-bunch type genotypes; spot 4 was present in these accessions with lower concentration. The polymorphic patterns revealed on 2-D PAGE could be used to differentiate subspecies *fastigiata *(Spanish type) (Fig. 2) and subspecies *hypogaea *(runner type) (Fig. 3).

### Polypeptide sequence analysis

**Figure 4:**
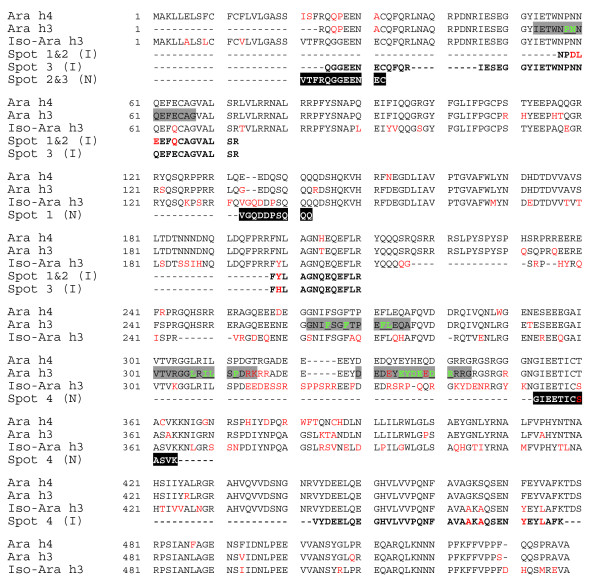
**Amino acid sequences alignment**. Amino acid sequences alignment of peptide sequences (N = N-terminal sequences; I = internal sequences by using in-gel trypsin digestion and sequencing), in bold-faced, of spots 1–4 with the published peanut allergen sequences of *Ara *h4 (AAD47382), *Ara *h3 (AAC63045), and iso-*Ara *h3 (ABI17154) (26). Sequences obtained by N-terminal sequencing are shaded in black. The different amino acid residues are colored in red. The amino acid sequences of *Ara *h3 IgE-binding epitopes [24] are shaded in gray and the critical amino acids to IgE binding are colored in green and underlined.

Protein peptide sequence analysis was conducted. The four polymorphic protein spots 1–4 were excised from the 2-D gels and PVDF membranes for peptide sequencing. For internal sequencing, two to three peptides were randomly picked and sequenced from each spot after in-gel trypsin digestion. The internal and N-terminal peptide sequences obtained for each spot and their homology identified through database searches are summarized in Table 2 and Fig. 4. All peptide fragments had significant sequence homology to known peanut allergens, *Ara *h3, *Ara *h4, and iso-*Ara *h3 [13] (Fig. 4). Interestingly, all amino acid sequences of these 4 spots in Fig. 2 and Fig. 3 are present in different regions of peanut allergen proteins as aligned with the published peanut allergen sequences (Fig. 4).

Peptide sequence of spot 1 was unique, and present only in Spanish-type peanuts. Two peptides sequenced after in-gel trypsin digestion were the same, while one fragment gave 100% (FYLAGNQEQEFLR) identity and another fragment gave 88% (14 out of 16 amino acids) identity with iso-*Ara *h3. The N-terminal sequence (VGQDDPSQQQ) of spot 1 was 100% identical with iso-*Ara *h3, whereas *Ara *h3 and *Ara *h4 have two amino acids missing in this region (Fig. 4). N-terminal sequencing for spot 2 and spot 3 resulted in the sequences containing VTFRQGG, identical with the sequence for iso-*Ara *h3 [13]. The N-terminal sequence of spot 4 was GIEETICSASVK, 100% identical with iso-*Ara *h3 and one amino acid (S/T) different from *Ara *h3 and *Ara *h4, supporting that spot 4 is the C-terminal part of this protein which always starts with GIEETIC [13].

## Discussion

The initial intention of this study was to profile the storage proteins using improved protein extraction method and to identify protein markers that could be used to separate subspecies of peanut, such as *hypogaea *and *fastigiata*, in order to select diverse breeding lines for mapping population construction. Based on the preliminary protein profiles [14], we selected Tifrunner and GT-YY20 for development of recombinant inbred lines (RILs) for genetic mapping. On 2-D PAGE gels, several proteins, labelled as spots 1–4 with similar molecular mass and different p*I*s, were sequenced. The peptide sequences obtained from these spots were all aligned to peanut allergens, such as iso-*Ara *h3 (AAT39430), indicating that this single gene encoded protein may be processed differently in different peanut subspecies. The partial cDNA sequence (accession number AY618460) was deposited in GenBank by Kang and Gallo-Meagher [15] in 2004. A full-length cDNA sequence identified in our EST sequencing project has been submitted to GenBank (DQ855115). The internal and N-terminal sequences of peptide spot 1 suggest that the apparent rearrangement of the amino acid sequence has occurred (Fig. 4).

In peanut the majority of seed storage protein (about 87%) is globulin consisting of two major fractions, arachin and conarachin [16]. The arachin subunits consist of the acidic polypeptides and the basic polypeptides [17]. The uniformity of the one-dimensional SDS-PAGE protein profiles within the runner type and Spanish type cultivars and breeding lines is in agreement with the studies [7-9], indicating that very low variation in protein profiles was detected in cultivated peanut using SDS-PAGE gel electrophoresis.

Generally, SDS-PAGE is not a sufficiently-powerful technique to distinguish a specific cultivar. Therefore, we adopted the widely used protocol developed by Damerval et al. [18] and introduced some modifications including a preliminary de-fatting step of peanut seeds for 2-D PAGE separation. We were able to generate 2-D electrophoresis gel separations with superior resolution and recovery from peanut seeds. Bianchi-Hall et al. [9] reported that the polypeptides of acidic arachin using SDS-PAGE distinguish Spanish from other market type cultivars. In this study, we did not identify the four bands in the range of acidic arachin by SDS-PAGE (Fig. 1), but we could detect the fourth spot of protein on 2-D PAGE for Spanish type genotypes (Fig. 2). We also detected a 26 kDa polypeptide by SDS-PAGE; this polypeptide could be used to differentiate Spanish and runner.

## Conclusion

This study demonstrated that two-dimensional electrophoresis (2-D PAGE) achieved a better resolution of protein profiles of peanut seeds, revealing polymorphisms between runner and Spanish genotypes. The basic arachin group, having one heavy band on SDS-PAGE gels at about 22 kDa, was resolved into several spots or subunits on the 2-D PAGE with distinct isoelectric points and slight differences in molecular weights. These proteins are isoforms (iso-*Ara *h3) of each other and the iso-allergens may be modified by post-translational cleavage. These results suggest that there may be an association between these polymorphic storage protein isoforms and peanut subspecies *fastigiata *(Spanish type) and *hypogaea *(runner type). Future studies could be designed to test the allergenic reactions of these peanut genotypes with different protein profiles and association with the resistance to aflatoxin contamination [19].

## Methods

### Plant materials

Twelve peanut genotypes were used in this study. There were six runner-type peanut genotypes: Georgia Green, A100, A104, GK7, A13 and Tifrunner, and six Spanish-bunch type peanut genotypes: ICGV 95435 (International Crops Research Institute for the Semi-Arid Tropics, Patancheru, India), MXHY and ZQ48 (Chinese landraces), and GT-YY20, GT-YY7 and GT-YY79 (Spanish derivatives with runner type peanut in their pedigrees, obtained from Crops Research Institute, Guangdong Academy of Agricultural Sciences, China). To avoid the effects of different locations, all genotypes were grown in Tifton, GA in 2003. Seeds were harvested at full maturity per normal production practices. After harvest, seeds were air-dried at 40°C and stored at 4°C before use.

### Total protein extraction

The total protein extraction was modified from TCA/Acetone protein extraction protocol [18] with the first step of de-fatting using hexane. Dry peanut kernels (20 g) of each genotype were frozen in liquid nitrogen and ground to powder in a mill and defatted with hexane (10 ml/g dry weight) at -20°C overnight. The defatted samples were collected by centrifugation (15,000 × g for 10 min at 4°C), air-dried, and ground to a fine powder in a pre-chilled mortar and pestle in liquid nitrogen. Protein extraction and precipitation were performed in 10% (w/v) trichloroacetic acid in cold acetone with 0.07% (v/v) β-mercaptoethanol at -20°C for 2 h, followed by centrifugation at 10,000 × g for 10 min at 4°C. The pellets were washed twice with cold acetone containing 0.07% β-mercaptoethanol, followed by washing twice with cold 80% acetone and then centrifuged at 10,000 × g for 10 min at 4°C. The pellets were air dried and stored at 4°C overnight. The total proteins were dissolved in lysis buffer (10 μl/mg) containing 9.5 M urea, 4% Igepal CA-360 (Sigma, St. Louis, MO), 2.5% ampholytes (0.5% pH 3.0–10, 0.5% pH 4–6, and 1.5% pH 6–8) (Sigma), 5% β-mercaptoethanol, and kept at 35°C for 30 min. After centrifugation (15,000 × g, 20 min, 25°C), the supernatant was collected for loading in first-dimension gel electrophoresis, or alternatively, for storing at -20°C until use. The supernatant protein concentration was determined using the Bradford [20] assay. The experiment was conducted twice, and each genotype was run at least three times.

### SDS-PAGE and two-dimensional PAGE electrophoresis

Total protein samples from these twelve peanut genotypes were first profiled using SDS-PAGE (15% separating gel with 4% stacking gel) according to the method of Laemmli [21] with the Mini-PROTEIN ^®^II Dual Slab Cell System (BIO-RAD, Hercules, CA) [22]. Total proteins (100 μg) from each sample were loaded onto SDS-PAGE gels. Low-range protein markers (Sigma) were used as molecular mass standard. The gels were electrophoresed (120 V, 1.5 h), stained with 0.125% Coomassie blue R-250 in 40% methanol and 10% acetic acid. For 2-D PAGE, total seed proteins (1 mg) were loaded into tube gels (8 M urea, 4% acrylamide, 2% Igepal CA-630, 0.5% ampholyte pH 3.0–10, 0.5% ampholyte pH 4–6, 1.5% ampholyte pH 6–8, 0.01% ammonium persulfate, and 0.1% TEMED), and overlaid with 20 μl sample overlay buffer (4 M urea, 0.25% ampholyte pH 3.0–10, 0.25% ampholyte pH 4–6, 0.75% ampholyte pH 6–8, 2.5% β-mercaptoethanol, 1% Igepal CA-360, and 0.05% Bromophenol blue). Isoelectric focusing (IEF) was conducted by using Mini-Protean^® ^2-D Electrophoresis Cell (BIO-RAD). The upper and lower chamber buffers were 100 mM NaOH and 10 mM H_3_PO_4 _respectively. IEF conditions were 200 V for 15 min, 300 V for 15 min, 400 V for 30 min, and 750 V for 6 h. The focused tube gels were equilibrated immediately for 30 min in 10 ml SDS equilibration buffer (60 mM Tris-HCl, pH6.8, 2% SDS, 10% glycerol, and 0.05% Bromophenol blue), or kept at -20°C until use. After equilibration, the tube gels were embedded in a 1% agarose solution at the top of the 2-D gel. The second dimension was run on 15% polyacrylamide-SDS gels in a Mini-Protean^® ^3 Cell (BIO-RAD), 120 V for 90 min. The gels were stained with Coomassie Brilliant Blue R250 and all gels were scanned and the spot intensities were analyzed using the software Image Master-2D (BIO-RAD). The interesting spots of seed protein among the genotypes were identified by gel-to-gel comparison. For molecular weight determination, low molecular weight standard (Sigma) was used.

### Peptide sequencing

Protein peptides were excised from the 2-D gels and PVDF membranes for peptide sequencing using electrospray ionization tandem mass spectrometry (ESI-MS/MS) to obtain internal peptide sequences and using the conventional Edman degradation method to obtain N-terminal sequences. Protein spots from the gels were excised with combined total protein amount up to 10 pg, and were subjected to in-gel digestion and analysis by ESI-MS/MS to obtain peptide sequence information at the Protein Chemistry Core Facility, Baylor College of Medicine (Houston, TX). When peptide sequences could not be obtained unambiguously by using ESI-MS/MS, Edman degradation was performed using an Applied Biosystems Procise cLC sequencer to obtain sequence information for protein identification.

### Electrobloting and N-terminal sequence

To prevent *N*-terminal blockage during second-dimension gel electrophoresis, gels were poured at least 24 hr prior to running and 0.1 mM thiodiglycolate was added as a scavenger in the upper running buffer. 2-D gels were equilibrated for 30 min in 25 mM Tris, 192 mM glycine, 10% MeOH (pH 8.3), and then electroblotted to Immobilon-p PVDF-membrane (Millipore, Bedford, MA, USA) at 300 mA for 4 hr in a Mini Trans-Blot^® ^Electrophoretic Transfer Cell (BIO-RAD). The membrane was subsequently equilibrated for 5 min in deionized water and proteins stained with 0.05% Coomassie Blue in 1% acetic acid and 50% methanol for a few min, destained in 50% methanol until background was pale blue. The membrane was rinsed for 5–10 min in deionized water and air-dried. Spots were excised and used for *N*-terminal amino acid microsequencing at Baylor Medical School (Houston, TX).

### Database sequence homology analysis

Internal and *N*-terminal peptide sequence homology identification was performed using basic local alignment search tool (BLAST) [23] against known or translated open reading frames of expressed sequence tags (ESTs) in the databases at the National Center for Biotechnology Information (NCBI) and SWISS-Prot.

## Authors' contributions

XQL performed the experiments and wrote the first draft of the manuscript. ML performed the sequence search and CCH provided plant materials. BZG conceived the research and revised the manuscript. All authors read and approved the final manuscript.

## Figures and Tables

**Table 1:** List of cultivated peanut used in this study

Cultivars	Subspecies	Market type	Origin
GT-YY20	*fastigiata*	Spanish	China
GT-YY79	*fastigiata*	Spanish	China
GT-YY7	*fastigiata*	Spanish	China
ZQ48	*fastigiata*	Spanish	China
MXHY	*fastigiata*	Spanish	China
ICGV 95435	*fastigiata*	Spanish	India
Georgia Green [25]	*fastigiata*	Runner	USA
A100	*fastigiata*	runner	USA
GK7	*fastigiata*	runner	USA
Tifrunner	*fastigiata*	runner	USA
A104	*fastigiata*	runner	USA
A13	*fastigiata*	runner	USA

**Table 2:** Internal peptide and *N*-terminal sequences of some protein spots of cultivated peanut

Spot^1^	Internal peptide sequence	N-terminal peptide sequence
1	FYLAGNQEQEFLR NPDLEEFQCAGVALSR	VGQDDPSQQQ
2	FYLAGNQEQEFLR NPDLEEFQCAGVALSR	VTFRQGGEENEC
3	QGGEENECQFQR FHLAGNQEQEFLR IESEGGYIETWNPNNQEFECAGVALSR	VTFRQGGEENEC
4	AQSENYEYIAFK VYDEELQEGHVLVVPQNFAVAAK	GIEETICSASVK

^1^Spot numbers are corresponding to the spot numbers in Fig. 2.
